# The Choice of General Anæsthetics in Surgery and Obstetrics*This was the title of the subject for general discussion before the Medical Society of Virginia, in session in Richmond October 10, 1887. Dr. McGuire was the leader.

**Published:** 1888-02

**Authors:** Hunter McGuire

**Affiliations:** Ex-President American Surgical Association, Ex-President and Honorary Fellow Medical Society of Virginia, etc., Richmond, Va.


### CULLINGS FROM CONTEMPORARIES.

### THE CHOICE OF GENERAL ANÆSTHETICS IN SURGERY AND OBSTETRICS.*

*By Hunter McGuire, M. D., LL. D., Ex-President American Surgical Association, Ex-President and Honorary Fellow Medical Society of Virginia, etc., Richmond, Va.*

[We reproduce the following article, from the *Virginia Medical Monthly*) because we consider it not only interesting reading, but an able review of the subject of anæsthetics, from the standpoint of a large personal experience and intelligent observation, by one well fitted to weigh facts and make deductions; and so situated as to have unusual facilities for observation.

The subject is of so much importance, and generally, so little understood in all its bearings, that we believe we do our readers a service in presenting it here. In so doing we ask to be pardoned the liberty of saying that with regard to the use of chloroform in military surgery our own experience corroborates Dr. McGuire's statements. In the hospitals and on the field, chloroform was used with a freedom bordering on reeklessness; it was given in all operations, and with the utmost freedom, and (it must be born in mind)—it was not—always,—very rarely, indeed—"Squibb's chlo-

*This was the title of the subject for general discussion before the Medical Society of Virginia, in session in Richmond October 1[illegible], 1887. Dr. McGuire was the leader.

roform;"—there was no guarantee of its purity, but, on the contrary, no doubt, much was used that was *not* pure,—yet the occurrence of an accident under its use, was *rare*, and a death from its administration, or, during its administration was unknown, in many hundreds of cases. Hence, quite, naturally, our predeliction is for chloroform, in general surgery. We cannot help believing, in view of our own experience in military surgery, and a somewhat extended observation in private practice since the war,—especially as other surgeons—amongst them Dr. McGuire—testify to the same effect, that the dangers of choloroform anæsthesia are greatly exagcrated.—Ed.]

Mr. President—I believe the members of this Society will generally concur in the wisdom of the Committee in selecting for discussion to-day the subject announced, "The Choice of General Anæsthetics in Surgery and Obstetrics."

For forty years medical journals and books have been rich in articles on the different general anæsthetics. Papers almost innumerably have been written on nitrous oxide, chloroform, ether, bichloride of methylene, and five or six other agents—all except the first belonging to the class of alcohols or ethers; but comparatively very few discussions had been hear, or articles written, upon the relative value and dangers of the different anæsthetics. What anæsthetic to use is an important, often vital question, and to most of us one of daily occurrence, but it is still undecided. The two principal agents, chloroform and ether, have been in use for many years—ether since 1846, and chloroform since 1847—but the profession is undetermined which is the safer and better. I may say in passing, but without any intention of discussing the idea, for it has, I think, been definitely settled as erroneous, some writers have stated that the use of any anæsthetic lessens the success of operative sargery.

In France, except in the city of Lyons; in Germany and Austria, except in the city of Vienna, chloroform is almost exclusively given. In Italy chloroform is generally preferred. In Great Britain chloroform, and mixtures of chloroform with other anæsthetics are principally employed; probably about one-third of the administrators use ether, aad some nitrous oxide alone, or combined with ether. Dr. H. W. Boone, of Shanghai, informs me that chloroform is the agent principally given in the Northern and Western States, and in the Southərn States chloroform is chiefly administered. From this it will be seen that chloroform, by itself, or combined with other anæsthetics, is much more frequently used than ether. How much more frequently we have no means of accurately determining, but taking obstetrical cases along with the surgical, it may safely be said that to-day, throughout the civilized world, chloroform is given twenty times where ether is administered once. The questiou as to the relative advantages and disadvantages of these two agents can only be eventually disposed of by the combined experience of many observers. The experience of no one man, or no small set of men, can determine the question; and until associations like this scientifically investigate and answer it, some will give ether, some chloroform, some nitrous oxide, bromoform, bromide of ethyl, etc., as their custom, fancy or convenience may decide.

Neither your patience nor the time allowed by the Committee for this paper, and the discussion to follow, will permit me to attempt an exhaustive essay on this subject, and I propose to restrict what I have to say to the relative value and danger of the two principal general anæsthetics in use, chloroform and ether. I shall confine my remarks chiefly to what I have myself observed in the almost daily use of one or both of these agents. I hope in this way to open the discussion, and to elicit the personal experience of members of this body.

It may be suggested that in the rapid advance and progress of science, it is probable some other anæsthetic, safer and better in every way than either chloroform or ether, will soon be discovered, and the discussion of the relative value of these agents become unnecessary; but we must remember that many years of very patient observation will be needed to settle the fact that some new agent is safer and better, and that we of this age and generation will not likely live to see it. I venture to predict that when the experience of the civilized world is collected and analyzed, it will be found, if indeed it is possible to definitely settle such a question, that in certain cases ether should be given, and in certain other cases chloroform employed, and that every good surgeon will be expected to exercise discrimination in the selection of his anæsthetic. For myself, I am wedded to neither of these agents. In general terms, in feeble, very anæmic people, or those sufferring from the prostration of shock, or loss of blood, I prefer ether; in either the young or the old, or in cases when cardiac, renal or pulmonary trouble is suspected, as a rule, I think chloroform is safer. Accidents occasionally follow the administration of both ether and chloroform, this, too, in the hands of competent and most skillful men. Already between four and five hundred deaths from chloroform have been reported, and nearly if not quite one hundred deaths from ether. What the ratio of either of the agents is to the number of inhalations, we are so far unable to determine. That both agents sometimes kill the patient, the most bigoted and partizan advocate of either ether or chloroform must admit. But which one of the anæsthetics is more dangerous and apt to kill is the paramount but undetermined question. Safety of the patient is the important point, before which all else should yield; compared to that, convenience, comfort, time, money and everything else are as nothing.

The tracings of the sphygmograph show invarible during the inhalation of chloroform, marked depression of the heart and circulation. In anæsthesia from ether, this is only occasionally seen, and then it is not so marked. The spygmograph shows that depression of the vaso-motor functions and cardiac paralysis is more likely to occur from chloroform than from ether; that the former is much the more powerful and dangerous agent; but clinical experience shows that when the vapor of chloroform is withdrawn, and consciousness returns, the patient is free from all danger from the anæsthetic. In ether, several minutes after the vapor is taken away, and after all danger from anæsthetic is supposed to have passed, when all ether vapor we would think had escaped from the lungs, dangerous symptoms suddenly present themselves, from which the patient is with difficulty rescued, or even death itself takes place. Or again, hours or even days after ether has been given, acute nephritis or pneumonia, directly traceable to the ether, occurs, threatening the life or causing the death of the patient. We may say, then, that chloroform is the more powerful and immediately dangerous anæsthetic; that when it kills it does so suddenly, shockingly; but that when the vapor is withdrawn and consciousness begins to come back, the danger is absolutely over. Ether may kill as chloroform does, just as suddenly, during the operation, but tt is much less likely to do so. The danger from this anæsthetic is, however, not over when the vapor is removed ; alarming symptoms or death may occur, by cardiac paralysis, a few minutes after, or by acute nephritis or pneumonia, hours or days after the administration. I think with regard to the selection of an anæsthetic this much is established, that in acute or chronic diseases of the kidneys or lungs, ether is more dangerous as an anæsthetic than chloroform.

I spoke only of paralysis of the heart in connection with chloroform, because it is the most common mode of death in fatal cases, probably more common than all the other causes combined ; but we all know that this is not the only mode of death from either of these anæsthetics. Both ether and chloroform, especially the latter may kill by using too concentrated a vapor ; both may kill during the period of muscular excitement, or by paralysis of the respiratory nervous centre.

In selecting the anæsthetic, I am somewhat governed, other things being equal, by the character of the assistant who is to administer the narcotic. If he is experienced or skillful, or nervous, and disposed to attend to the duties of every one around him and neglect his own, ether is the safer. To give chloroform requires skill, and the ability not always possessed by an assistant, of attending strictly to his own business. A man accustomed to give ether is not always a safe administrator of chloroform. To ask a patient to take long, deep or rapid inspirations of chloroform vapor is very dangerous. The greatest danger from this agent is in the early stage of its administration, long before unconsciousness occurs, when, by a too concentrated vapor, or by its too rapid administration, the nervous centres presiding over the heart and circulation may be surprised and overwhelmed. And when a patient in pain, or in dread of the knife being used before unconsciousness takes place, breathes rapidly and deeply in order that he may more quickly become nacrotized, it is safer to partially or for a time completely remove the chloroform. I think it safer, too, when practicable, to keep the patient's head turned to one side in using chloroform, so that the vapor, four times heavier than air, may not occupy the base of the cone and exclude the atmorphere. In giving chloroform it is better to begin with a small quantity and allow with the vapor plenty of fresh air, and gradually accustom the patient to its use. Never give chloroform in a hurry.

The use of ether does not demand so much care, although, if what I have stated with regard to its dangers is correct, a certain amount of skill and caution should be observed. This, after using it very often, is the impression I have of it; but in a Northern city, where, in the opinion of the profession, to give chloroform is almost a penal offence, I saw ether administered with a recklessness which astonished and alarmed me, and I lost my interest in the operation being performed in watching the purple, asphyxiated patient, and wondering why he did not die; and why he did not, I confess, was beyond my comprehension. I understood then why men who gave ether in that way were, and well might be, afraid of chloroform.

The plan of giving alcohol, as a heart stimulant, just before administering chloroform, is, in my opinion, liable to serious objections, and for many years I have abandoned its use in this way. In the first place, it is difficult to know in all cases what a stimulant dose of whiskey is, so much depends upon the habit and temperament of the patient, or the depression which disease, injury or mental apprehension has produced. A stimulant dose of whiskey in one man may be a sedative dose in another, and in all cases the secondary sedative effect of the alcohol may come on, especially if the anæsthesia is kept up for some length of time, and add to, rather than prevent the depression. I am satisfied that alcohol increases the duration of violence of the stage of excitement, and makes nausea and vomiting more likely to occur during and especially after the operation is completed. We all agree that men accustomed to the free use of liquor are bad subjects for anæsthetics, and my observation leads me to think that a dose of whiskey before giving chloroform averts no danger, but rather adds to this, as well as to the general discomfort of the patient.

I was surprised to see, a few days ago, a paper on chloroform, written by a very deservedly eminent surgeon of New York, in which he advocates giving a very small dose of chloroform, say 20 or 30 drops, to be administered in a concentrated vapor. If alarming symptoms set in, this amount of vapor could be speedily pumped out of the lungs by artificial respiration. This is dangerous doctrine to teach, and, coming from so prominent a man, calculated to mislead others. In the only death of chloroform that has happened in my hands, the action of the heart stopped instantly. I was given the vapor myself; had my finger on the woman's pulse, and the first indication of danger was the sudden stoppage of the heart. It did not flutter, or intermit, or grow weak, but abruptly ceased, and this while the patient was yet conscious.

Although all means were tried to resuscitate the patient, I knew when it happened that no power on earth could ever make that heart beat again. It was like the syncope of concussion of the brain; the contractile pewer of the heart was annihilated; and I am sure that the advocate of small doses of concentrated vapor of chloroform will find, when disaster comes, that he may remove from the lungs all of the vapor by artificial respiration; but he cannot in this way take away the impressiou he has produced on the nerve centers, which have stopped the heart's action. He may as well say he will not apply a blazing torch to the fuse which leads to a magazine of powder, but that he will use only a spark of fire, and if danger comes, he can blow that out.

There is one hazzard from chloroform which, although we are frequently taught we are apt to forget—that is, operating during partial anæsthesia. I believe we are all liable to forget this great and unjustifiable danger of beginning or performing an operation before the patient is fully under the anæsthetic. Many of the deaths from chloroform have happened in this way. A tooth is to be drawn, a pile tied, a felon opened, or some operation performed which is the work of a few seconds; and the inclination is to operate before the anæsthesia is complete. It is extremely hazardous to do this. Possibly the brain has not lost consciousness, and the patient is dimly aware that the operation has begun, that the pain he dreads is to follow, and in consequence of his terror, fatal syncope comes on; or, if intellectual consciousness is lost, there seems to be—if I may so speak—a consciousness still left in the nerve centers, which preside over the heart and circulation, and the impression of pain on them is fatal. Ether is safer when the operation is to be performed with partial anæsthesia.

In all operations about the face or throat, where blood or other fluids may escape into the wind-pipe, ether is the more dangerous, and chloroform the safer agent to use. I do not think I ever saw the irritability of the larynx or trachia entirely lost in anæsthesia from chloroform, but I have seen this happen in other cases; the sensibility or reflex irritability is for the time abolished, and foreign substances easily find their way into the wind-pipe. Possibly the cold vapor of ether may to some extent account for this fact.

In the beginning of this paper, I said that I thought chloroform the safer agent in cardiac troubles. I wish to except from this class a nervously weak heart. In organic valvular disease of the heart, with the usual compensative muscular hypertrophy, I have given chloroford hundreds of times, and never had cause for alarm. On the contrary, the heart's action became usually more quiet and regular, and chloroform is safer here than ether. But in a heart weak from fatty degeneration, or from loss of blood, or great anæmia from other causes, any anæsthetic is hazardous, but chloroform, I believe, is more dangerous than ether.

Of all the elements of danger, to my mind, from chloroform, fear on the part of the patient is the greatest. If the patient is, so to speak, in mortal terror of the anæsthetic, the heart is nervously weak, and the hazard to life is especially great. All things being equal, ether, then, is the safer agent to use. To the dread of the operation the patient may have added the hazard of the anæsthetic, and the emotional condition be one of absolute terror. Fatal cases under such circumstances are not uncommon. A few words of encouragement from the administrator, or a calm, confident manner on his part, may allay the anxiety; but if it does not, and the great alarm continues, it will be safer to give hypodermically ¼ grain of morphia and 1-100 of a grain of atropia, and wait fifteen or twenty minutes for the physiological effects of these drugs before giving the anæsthetic. Emotional excitement greatly increases the chances of paralysis of the nerve centers which preside over the circulation. Morphia obtunds the sensibility of the nervous system, and at the same time is a cardiac stimulant. Atropia is probably still more powerful in this direction, and the employment of these drugs under such circumstances will lessen or completely remove the danger. I think both reason and clinical experience have confirmed this fact.

I am sure that the emotional excitement of fear has not had its due weight with operators. It is not altogether possible to explain its physiological effect upon the nervous system, and the heart and circulation. It varies so much with the sensibility or emotional nature of the individuals. With some it is fixed, and impossible to allay; with others it is transient, and easily removed; with some its effect upon the nervous system is profound, lasting, and dangerous; with others this impression is trifling and evanescent. It is impossible always to estimate its power or effect; but that it is an important element of danger all observant administors will admit.

Children take chloroform well and safely. I believe the most zealous advocates of ether confess that chloroform is safer and better for them. The principal reason for its safety in children is that they are ignorant of its danger, and are not afraid of being killed by it. How else can we satisfactorily explain the comparative immunity from danger to the young?

It is a significant fact, too, that Nussbaum has seen in military life 40,000 administrations of chloroform without an accident; and that in the Confederate Army Corps to which I was attached, as Medical Director, chloroform was given 28,000 times without a death ascribed to its use. Can these facts be explained by the age, sex, health, etc., of the soldiers. I think not; because men of the same age, health, etc., sometimes die in the hands of civil surgeons in chloroform anæsthesia. In military life, I know, not simply from theory, but from actual observation, that the pain of a gunshot wound, and the danger from it to life, or loss of limb, makes the soldier dread the hazard of chloroform very little—if at all.

It is a significant fact, too, in this connection, that chloroform has been given to hundreds of thousands of women in child-birth; and when the agent was in the hands of competent men, but one fatal case has occurred, and in this instance it was by no means certain that death was due to the anæsthetic. Even when surgical operations have been required and performed in obstetrical cases, no deaths have occurred from its employment. Indeed, so far as we can see from the experience of thousands of cases. chloroform is absolutely safe in parturient women, even less dangerous than a dose of ergot, or oil, or opium. How can we explain this great and entire exemption from danger when using chloroform in obstetrical cases? We cannot account for it by the sex of the patient, or the small quantity given at a time, for in other conditions death sometimes results from small doses. Her age has nothing to do with it. She is likely stronger and more healthy during the child-bearing period of life than in youth or old age; nor does this same condition of age, or strength, or health, avert the danger from chloroform in other cases not obstetrical. The recumbent position surely does not explain it; for while this position in anæsthesia, from both chloroform and ether, should, when possible, be always observed, many deaths from chloroform and ether have happened to patients in the recumbent posture. Have the pains of labor anything to do with this exemption? I believe not; for we have pain from the surgeon's knife, and from the disease or injury for which the operation is performed. The pains of labor too, often stop for several hours. I think we can only explain this absence of danger from chloroform in obstetrics by the absence, on the part of the patient, of any dread of the chloroform. As a rule, so far from any fear of it, she begs piteously for it, and her condition renders her absolutely free from that emotional state which depresses the heart and circulation.

I would like to speak, if time permitted, of the importance of using pure articles of chloroform and ether; of giving first ether, as I have sometimes done, in cases of functionally weak hearts, and substituting chloroform before anæsthesia is complete; of changing the anæsthetic when one is found to act badly, or dangerous symptoms arise; and several other points in regard to the selection of anæsthetics; but I forbear. I hope some of these topics will come up in the general discussion of the subject.

Before closing this paper, I am sure the Society will unite with me in deploring the sometimes bitter and partisan debates so often seen in discussing the question of the relative value of ether and chloroform. Some, who give exclusively one of these anæsthetics, will scarcely tolerate the discussion of the comparative safety and value of the other. Statistics, general facts, and clinical experience have no weight with them, and denunciation takes the place of argument. Some who give chloroform say in effect, unjustly I know, that the administrators of ether are wanting in moral courage, and are afraid of public opinion; that they would rather subject their patients to the hazards of the after-consequents of ether than the immediate dangers of chloroform; that the latter is less frequent, but when it does occur, communities are more shocked and frightened by it. Some of those who give ether, as bitterly and vehemently denounce the men who give chloroform. In the last text-books on surgery, issued this year, you will find the following: "In general, there is no comparison between these agents; ether is so much safer than chloroform that the latter is fast disappearing in practice. The estimated death rate after ether is 1 in 20,000; in chloroform, 1 in 3,000; seven lives are sacrificed to chloroform to one by ether." Such statements are the outcome of prejudiced brains, and absolutely unwarranted by any facts or figures known to the profession. In the discussion of a purely scientific question, the proper solution of which may involve human life, such feeling should be avoided. Let those who prefer one of these agents, give to his fellows who select the other, credit for equal honesty, and the same desire to save human life and suffering. Surely the bigotry and intolerance we unfortunately sometimes see in theological and other debates, and the partisan rancor often found in political contests, should have no place in questions like the one under consideration.—*Virginia Medical Monthly.*

---

THE WAY TO AID A HOME JOURNAL.—There are a few physicians in this State who pay their subscription to DANIEL'S TEXAS MEDICAL JOURNAL from two to five years in advance. We name Dr. R. M. Swearingen, Dr. T. D. Wooten, Dr. R. T. Knox, Gonzales, 1st Vice-President T. S. M. A., Dr. Jos. Getzwiler, Goliad, and Dr M. D. Sterrett, of Grand Bluff; while *many* interest themselves and aid us by getting up clubs.

---

DR. A. E. HOLDEN, representing Fairchild Bros. & Foster—the manufacturers of Peptogenic Milk Powders, Pancreatine, Fairchild's Pepsine and other well known and standard Pharmaceuticals, favored us with a visit last week. He is an enthusiast in the cause of artificial digestion, and takes pleasure in demonstrating the virtues of the Fairchild preparations. Mr. H. will canvas Texas, and we bespeak for him a kindly reception.

